# Increasing Retention in a Large-Scale Decentralized Clinical Trial: Learnings From the COVID-RED Trial

**DOI:** 10.1016/j.mcpdig.2025.100264

**Published:** 2025-09-09

**Authors:** Laura C. Zwiers, Duco Veen, Marianna Mitratza, Timo B. Brakenhoff, Brianna M. Goodale, Paul Klaver, Kay Y. Hage, Marcel van Willigen, George S. Downward, Peter Lugtig, Leendert van Maanen, Stefan Van der Stigchel, Peter van der Heijden, Maureen Cronin, Diederick E. Grobbee, COVID-RED Consortium

**Affiliations:** a Department of Global Health and Bioethics, Julius Center for Health Sciences and Primary Care, University Medical Center, Utrecht, The Netherlands; b Julius Clinical, Zeist, The Netherlands; c Department of Methodology and Statistics, Utrecht University, The Netherlands; d Optentia Research Programme, North-West University, Potchefstroom, South Africa; e Ava Femtec, Zürich, Switzerland; f Department of Environmental Epidemiology, Institute for Risk Assessment Sciences (IRAS), Utrecht University, The Netherlands; g Helmholtz Institute, Experimental Psychology, Utrecht University, The Netherlands; h Department of Psychology, Utrecht University, The Netherlands; i Department of Social Statistics, University of Southampton, UK

## Abstract

**Objective:**

To present retention strategies implemented in the coronavirus disease 2019 (COVID-19) rapid early detection trial, a decentralized trial investigating the use of a wearable device for severe acute respiratory syndrome coronavirus 2 detection, and to provide insights into study retention and investigate determinants of discontinuation.

**Patients and Methods:**

The COVID-2019 rapid early detection trial collected data from 17,825 participants from February 22, 2021 to November 18, 2021. Participants wore a wearable device overnight and synchronized it with a mobile application on waking. Retention strategies included common and personalized activities. Multivariable logistic regression was used to identify participants at high risk of discontinuation after 6 months in the trial. Results were combined with insights from behavioral theory to target participants with additional telephone calls.

**Results:**

Total of 14,326 (80.4%) participants remained in the trial after 6 months and 12,208 (68.5%) until the end of the trial. Multivariable logistic regression identified age, employment situation, living situation, and COVID-19 vaccination status as predictors of discontinuation. Subgroups at high risk of discontinuation were identified, and behavioral assessments indicated that the subgroup of vaccinated pensioners would receive additional telephone calls. Their dropout rate was 11.4% after telephone calls.

**Conclusion:**

This study describes how innovative and targeted data-driven retention strategies can be applied in a large decentralized clinical trial and presents the implemented retention strategies and discontinuation rates. Results can serve as a starting point for designing retention strategies in future decentralized trials.

With the increasing availability of digital technology influencing elements of clinical trials, decentralized clinical trials have gained much popularity.^1^ Decentralized trials can often quickly reach large populations, as they do not require physical site visits and follow more participant-centric approaches.^2^ The coronavirus disease-2019 (COVID-19) pandemic accelerated the adoption of decentralized trials, specifically in infectious disease research. Decentralized trials have the potential of collecting much real-world data but often suffer from high dropout rates,^3^, ^4^, ^5^ potentially due to difficulties with sustaining participant engagement without personal contact and low digital literacy among participants.^5^ High dropout rates hinder generalizability of trial findings, as the analyzed data may not be representative of the initially recruited population, and dropout may reduce power such that more participants need to be recruited to achieve a sufficient sample size. Moreover, as dropout could be related to participant characteristics, it may negatively affect the validity and reliability of trial findings.^6^ Decentralized studies conducted during the COVID-19 pandemic suffered from additional complexities, as the epidemiological context was constantly changing. Case studies from completed decentralized trials and insights into determinants of study discontinuation can inform strategies to boost retention. For example, identification of participants at highest risk of discontinuation can inform targeted engagement strategies toward those participants, which could increase study viability.

To date, there is limited evidence on determinants of retention in decentralized clinical trials.^7^^,^^8^ A cross-study evaluation found that referral by a clinician to the study, financial compensation, having the condition of interest, and age were associated with retention.^8^ In a trial of web-based smoking interventions, bonus incentives and the option to complete surveys through different modalities both increased retention,^9^ whereas another study found that nonmonetary incentives, such as notifications with study insights, were also considered rewarding.^10^ An interview study on participation in remote studies suggested that reminders for completing tasks and communications on the value of participants’ contributions are beneficial, while strategies that include gamification could feel patronizing.^11^ Finally, a large-scale, nonrandomized, remote COVID-19 surveillance study found that longitudinal study retention was highest among participants who were White, non-Hispanic, older, working remotely, and with lower socioeconomic vulnerability.^12^ In studies like the latter, performed within a constantly changing epidemiological context, participant retention may be additionally influenced by external factors and participants’ responses to these. For example, vaccination may reduce an individual’s perceived threat of the virus, which could lead to increased dropout from decentralized studies that aim to detect an infectious disease. Apart from evidence from previous studies, behavioral models may also inform expectations regarding compliance and participants’ response to incentives.^13^ For instance, additional reminders may boost retention among older participants but not among the younger.^14^

As retention plans and strategies are rarely published, sharing case studies of both successful and unsuccessful practices in decentralized clinical trials could contribute to the advancement of trial conduct, particularly in the absence of reporting standards for retention plans.^15^ To date, few publications on determinants of retention in decentralized clinical trials or strategies to maximize retention exist. In this paper, we describe the retention strategies implemented in the COVID-19 rapid early detection (COVID-RED) trial, which was a large decentralized clinical trial investigating the use of a wearable device for early detection of severe acute respiratory syndrome coronavirus 2 (SARS-CoV-2) infections in real-time during the pandemic. The protocol of this trial has been published.^16^

The objectives of this paper are twofold. First, we present the retention strategies implemented in the COVID-RED trial. Second, we provide exploratory insights into participant retention during the study. Specifically, we (1) investigate the study discontinuation rates, analyzed by participant characteristics; (2) identify which participants were at highest risk of discontinuation; and (3) assess the extent to which implementing an active engagement strategy with participants at higher risk of discontinuation improved retention.

## Patients and Methods

### Study Population

The COVID-RED was a single-blinded, 2-period, 2-sequence, randomized controlled trial that recruited and enrolled participants from February 2021 to June 2021. Participants were provided with a wearable device (the Ava bracelet; Ava AG) to be worn while sleeping and synchronized with a mobile telephone application on waking. In this application, participants were also asked to report any physical symptoms they experienced and factors potentially influencing their physiological parameters every day. The COVID-RED study investigated the ability of the device to detect SARS-CoV-2 infections compared with standard care, which was symptom reporting. The trial ran from February 2021 until November 2021 and comprised 3 phases. First, there was a learning phase of up to 3 months, after which there were two 3-month periods (period 1 and period 2) during which participants were randomly assigned to one of the 2 study conditions subsequently. In the experimental condition, data from both the wearable device and the daily symptom reporting were used to predict SARS-CoV-2 infections, and participants would receive an alert to get tested if these data indicated a likely infection. The control condition mimicked standard care, such that participants would receive an alert only if their daily symptom data indicated a likely infection. When receiving an alert, participants were advised to seek polymerase chain reaction testing for SARS-CoV-2 and asked to record the result in the application. Moreover, participants were asked to take SARS-CoV-2 serology tests periodically and fill out biweekly surveys. Further details on the study design have been published.^16^

### Study Recruitment

The COVID-RED trial aimed to enroll 20,000 participants from The Netherlands, with ∼13,000 from a normal risk population and 7000 from a high risk population. High risk individuals were those fulfilling predefined, self-reported criteria. Recruitment was done by inviting members of existing cohorts (Leidsche Rijn Julius Gezondheidscentra cohort,^17^ ParkinsonNext cohort^18^), as well as by public outreach campaigns and an advertisement campaign. Individuals interested in joining the study were referred to the COVID-RED website, which provided information on the trial’s background, aims, and procedures.

### Participant Characteristics

From February 22, 2021 to June 3, 2021, 57,161 individuals were screened, and 17,825 fulfilled inclusion criteria to be randomized. About 10,822 randomized participants were considered normal risk, and 7003 high risk. Total of 11,832 (66.4%) participants were recruited through social media, 843 (4.7%) through existing cohorts, 274 (1.5%) through a database of individuals interested in clinical study participation, 4873 (27.3%) through other sources, and for 3 (0.0%) the recruitment source was unknown. Table 1 presents the baseline characteristics.

**Table 1:** Baseline Characteristics of Participants

Variable	Number of participants 17,825 (100%)
Sex	
Male	5299 (29.7%)
Female	12,510 (70.2%)
Other or unknown	16 (0.1%)
Age	
Mean ± SD	46.4 ± 14.6
Median (IQR)	48.0 (35.0-57.0)
Education level	
Primary education only	157 (0.9%)
Lower vocational education	899 (5.0%)
Lower general secondary education	2741 (15.4%)
Higher general secondary education	1802 (10.1%)
Higher vocational education	3951 (22.2%)
Higher professional education	5328 (29.9%)
University education	2565 (14.4%)
No education	150 (0.8%)
Other education	232 (1.3%)
Employment situation	
Working at least 80% of full-time	8228 (46.2%)
Working less than 80% of full-time	3413 (19.1%)
Full-time housekeeper	805 (4.5%)
Full-time student	1222 (6.9%)
Pensioner	1746 (9.8%)
Job-seeker	468 (2.6%)
( Partially) incapacitated	1347 (7.6%)
Rentier	108 (0.6%)
Other	488 (2.7%)
Living situation	
Living together with partner	11,767 (66.0%)
In a stable relationship but living alone	784 (4.4%)
Single and living alone	2733 (15.3%)
Single and living with parents or children	1475 (8.3%)
Living with flatmates	1066 (6.0%)

Abbreviation: IQR, inter-quartile range.

### Retention Monitoring

After providing informed consent remotely and being randomized, participants were allowed to discontinue their participation at any time. A participant could be withdrawn for any of the following reasons: loss to follow-up, withdrawal of consent, death, or use of the Ava bracelet for purposes outside of the study (eg, menstrual cycle tracking). Retention was monitored on an ongoing basis throughout the trial. With limited available information on dropout rates in comparable studies, let alone those conducted during a pandemic, estimates were made of a dropout rate of 2.5% per month and a 75%-80% retention rate at study completion. The dropout rate was monitored weekly, overall, and stratified by study phase. In addition, missing data per data source were monitored.

### Planned Retention Activities

A retention plan was developed at the start of the trial to maximize engagement. The plan included biweekly Instagram posts, website posts, monthly newsletters, a designated helpdesk, and individual emails to request participants to take their serology tests. Some retention activities were based on participant behavior. For instance, participants who had been recruited but had not yet synchronized their bracelet received an email with tips for completing this task, and participants who had not taken their serology test by a certain date received reminders to do so.

In addition, a dynamic schedule to follow-up individual compliance through personalized emails was implemented during periods 1 and 2. This schedule comprised emails that informed participants of their compliance rate for the biweekly surveys (based on the last 3 surveys) and bracelet synchronization (based on the last 14 days). A gamification approach was adopted, with participants receiving a virtual medal (gold, silver, or bronze). The frequency of emails depended on compliance and could fluctuate over the course of the trial. Table 2 shows the interval between follow-up emails for participants with different compliance rates. Participants who confirmed their discontinuation from the study through the helpdesk or indicated they did not want to receive this communication, did not receive individual follow-up emails.

**Table 2:** Frequency of Follow-up Emails for Different Rates of Compliance in the Biweekly Survey and Bracelet Synchronization, the Cells Depict the Interval Between Follow-up Emails

Variable	Biweekly survey compliance			
	<25%	25%-50%	50%-75%	>75%
Bracelet synchronization compliance				
<25%	4 wk	4 wk	4 wk	8 wk
25%-50%	4 wk	4 wk	4 wk	8 wk
50%-75%	4 wk	8 wk	8 wk	8 wk
>75%	8 wk	8 wk	12 wk	12 wk

Another planned retention activity was the monitoring of participant activity. Participants who were inactive but did not indicate their discontinuation were considered non-active participants. Non-active participants were defined as those who (1) received the start pack but never onboarded, or onboarded but never synchronized the bracelet and submitted no serology tests to the central laboratory and completed no biweekly surveys, or (2) submitted no serology tests to the central laboratory and had 0% compliance on bracelet synchronization and biweekly surveys. Assessment of non-active participants was done once during period 1, after which non-active participants were contacted and given the chance to still start the trial. Those who did not respond were excluded from the trial.

### Data-Driven Retention Activities

At the end of period 1, higher than expected dropout rates were observed. Because period 2 was important for study analyses, additional targeted retention activities were implemented. Subgroups of participants at highest risk of discontinuation were identified. For those subgroups, participants were targeted in accordance with behavioral models.^13^^,^^19^^,^^20^ A plan was made to contact specific subgroups of participants at high risk of discontinuation through additional telephone calls. This additional activity was developed spontaneously during the trial on top of predefined retention activities given the observed dropout rates during period 1. Another reason for this ad-hoc retention activity was the intensified roll-out of COVID-19 vaccines (which was theorized might impact retention) and the summer holidays. An overview of all retention strategies by study period is shown in Figure.

**Figure:**
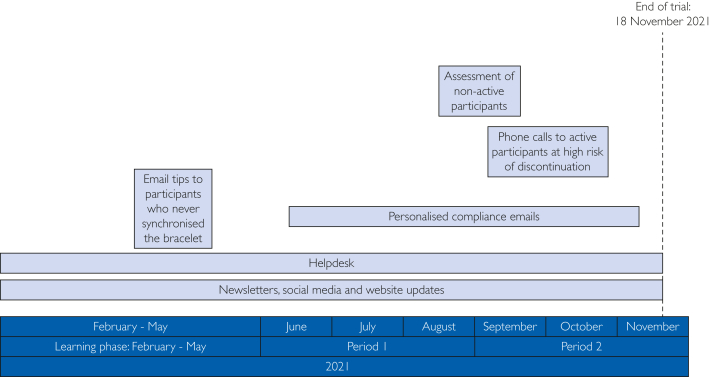
Overview of study periods and retention activities. Light blue panels represent general activities, while gray panels correspond to personalized activities.

### Data Analysis

Exploratory data analysis was done to provide insight into the discontinuation rate, subgroups at highest risk of discontinuation, and the impact of an active engagement strategy using telephone calls.

The discontinuation rate was calculated by dividing the number of participants who discontinued by the number of randomized participants. This was done per study period and stratified by participant characteristics.

Multivariable logistic regression was used to identify subgroups at high risk of discontinuation. The outcome variable was defined as a report of discontinuation to the helpdesk by September 1, 2021 (ie, the start of period 2). Determinants of interest were age (in years), sex (male, female, or other/unknown), education level, employment situation, living situation (see Table 1 for an overview of the possible categories of these variables), and vaccination status (vaccinated or not vaccinated). Significant predictors were selected for the final model based on a *P*-value below .05, and the final model included all first-order interaction terms.

Finally, the impact of additional telephone calls to participants at high risk of discontinuation was assessed. To decide which participants would receive a telephone call, the sample size of each subgroup at high risk of discontinuation was weighted with the theory-informed expectation of a higher dropout rate if follow-up activities would be intensified.^13^^,^^19^^,^^20^ The impact of this calling strategy was assessed in an exploratory manner by comparing the discontinuation rate at the end of period 2 with that before the start of period 2, and by comparing this rate with that of the full population. Potential confounding factors were not considered due to the lack of suitable confounder data.

## Results

### Time-On-Study and Discontinuation Rate

Of the 17,825 participants, 14,326 (80.4%) remained in the study until September 1, 2021 (ie, the start of period 2). Of the 3499 participants who dropped out before this date, 1355 dropped out during the first 3 months, and 2144 during period 1. Of the 14,326 participants who were still in the study at the start of period 2, 2118 (14.8%) still dropped out. 1884 of these dropouts happened in September, which was when non-active participants were excluded from the trial.

Stratified by sex, the discontinuation rate before the start of period 2 was comparable between women (19.4%) and men (20.2%). Total of 23.6% of participants older than 50 years dropped out before period 2, whereas only 16.6% of participants aged 50 years or under discontinued. Table 3 shows the dropout rate during the first 6 months of the trial stratified by level of education, employment conditions, and living situations. Dropout rates were generally comparable across groups, with some exceptions. For instance, pensioners and rentiers had higher dropout rates compared with those working (nearly) full-time (28.3% and 28.7% versus 16.8%).

**Table 3:** Dropout Rate During the First 6 Months of the Trial Stratified by Participant Characteristics

Variable	Number of participants	Number of participants who dropped out before period 2 (%)
Education		
Primary education only	157	36 (22.9%)
Lower vocational education	899	236 (26.3%)
Lower general secondary education	2741	583 (21.3%)
Higher general secondary education	1802	344 (19.1%)
Higher vocational education	3951	791 (20.0%)
Higher professional education	5328	990 (18.6%)
University education	2565	434 (16.9%)
No education	150	27 (18.0%)
Other education	232	58 (25.0%)
Employment		
Working at least 80% of full-time	8228	1380 (16.8%)
Working less than 80% of full-time	3413	679 (19.9%)
Full-time housekeeper	805	175 (21.7%)
Full-time student	1222	212 (17.3%)
Pensioner	1746	494 (28.3%)
Job-seeker	468	110 (23.5%)
(Partially) incapacitated	1347	311 (23.1%)
Rentier	108	31 (28.7%)
Other	488	107 (21.9%)
Living situation		
Living together with partner	11,767	2346 (19.9%)
In a stable relationship but living alone	784	147 (18.8%)
Single and living alone	2733	581 (21.3%)
Single and living with parents or children	1475	245 (16.6%)
Living with flatmates	1066	180 (16.9%)

### Determinants of Discontinuation

Multivariable logistic regression analyses were performed on randomized participants for whom data was complete at the end of period 1 (n=17,792). Participants were considered vaccinated if they had received any COVID-19 vaccination at that time (66.3%). The model, including all predictors of interest, identified age, employment situation, living situation, and vaccination status to be relevant (Supplemental Table 1, available online at https://www.mcpdigitalhealth.org/).

The relevant predictors and all first-order interactions were included in the final model (Supplemental Table 2, available online at https://www.mcpdigitalhealth.org/), which had an area under the curve of 0.697. Seven groups at higher risk and one group at lower risk of discontinuation relative to the reference category were identified using this exploratory method (Table 4).

**Table 4:** Groups of Participants at High/low risk of Discontinuation Identified in the Multivariable Logistic Regression Model^a^

Groups at high risk of discontinuation	OR^b^ (95% CI)	Number of participants
Full-time students who are single and live alone	1.104 (1.007-1.209)	121
Rentiers who are single and live alone	1.205 (1.006-1.443)	31
Rentiers who are single and live with their children/parents	2.619 (1.246-5.508)	1
Rentiers who live with flatmates	2.232 (1.297-3.843)	2
Pensioners who live with flatmates	1.230 (1.040-1.456)	24
Pensioners who have been vaccinated	1.087 (1.031-1.145)	1,279
Participants who are single and live alone and have been vaccinated	1.049 (1.015-1.085)	1,558
Groups at low risk of discontinuation		
Full-time students who have been vaccinated	0.945 (0.896-0.996)	590

a Abbreviation: OR, odds ratio.

b ORs are relevant to the reference category of individuals who work at least 80% of full-time, live with their partner, and are unvaccinated.

### Determination of Active Engagement Strategy

Across the groups at high risk of discontinuation, 3 subgroups were sufficiently large to proceed with the theory-based assessment for additional telephone calls. The first subgroup consisted of students who are single and live alone. Of 31 of the 121 participants had already dropped out, hence, there were 90 ongoing participants. No additional telephone calls were scheduled for this subgroup, as behavioral theory suggested that intensified follow-up would increase dropouts.^14^ The second subgroup comprised vaccinated participants who are single and live alone, with 1358 ongoing participants and 200 participants who have already dropped out. This subgroup was diverse in terms of other characteristics, and an active calling strategy was not expected to be beneficial for the subgroup as a whole.^21^^,^^22^ The final subgroup of interest included pensioned participants who had been vaccinated and consisted of 1019 ongoing participants, whereas 260 had already dropped out. This subgroup was targeted with additional telephone calls as it was hypothesized that the emotional value of the phone calls would serve as a motive for increased engagement to the relatively older individuals in this subgroup.^14^

Among the vaccinated pensioned participants, a special focus was given to those with 30%-70% compliance in wearing and synchronizing the Ava bracelet. For these participants, the most improvement could be expected because they were familiar with the bracelet but did not wear or synchronize it often enough. During the telephone calls, which aimed to retain participants in the trial, participants were thanked for their participation and asked about potential problems.

### Impact of Active Engagement Strategy

Of the 1019 ongoing participants in the subgroup of vaccinated pensioners, 79 (7.8%) had a 30%-70% compliance in the 14 days before the start of period 2 and therefore received a telephone call from the study helpdesk. About 9 (11.4%) of them dropped out during period 2. This dropout rate was lower than the dropout rate across the vaccinated pensioners before period 2 (20.3%) and also lower than the dropout rate in the full study population in period 2 (14.8%).

## Discussion

This study highlights the retention activities performed in a decentralized clinical trial during the COVID-19 pandemic and explores determinants of discontinuation. The overall dropout rate after 6 months was 19.6%, and stratifying dropout rates by participant characteristics showed that dropouts were more common among participants aged above 50 years and for pensioners and rentiers. An exploratory multivariable logistic regression model identified age, employment situation, living situation, and vaccination status as predictors of discontinuation. On the basis of behavioral theory, the subgroup of vaccinated pensioners was actively targeted with telephone calls with the aim of improving retention during the final study period. The dropout rate after these telephone calls was lower than the overall study dropout rate during this period, suggesting a potential positive effect.

Previous studies on determinants of discontinuation focused on incentives for participants to stay in the trial rather than personal characteristics.^8^, ^9^, ^10^, ^11^ To our knowledge, this study is one of the first to investigate various participant characteristics as potential drivers of discontinuation and to incorporate behavioral theory in the implementation of engagement strategies during the trial. By leveraging the expertise of behavioral scientists, we were able to target a subgroup that would potentially benefit from receiving additional telephone calls. Such personalized behavioral interventions are rarely used, whereas common strategies to influence behavior often do not work for all individuals.^22^ Future research could further optimize active engagement strategies by looking more closely at the performance of regression models that inform these strategies. In this study, the model discrimination and calibration were not considered when deciding on the active engagement strategy, but it could be of interest to look at these measures to decide whether the telephone calls would be worth the effort.

It was initially hypothesized that receipt of COVID-19 vaccination would be associated with higher risk of discontinuation, as it could affect participants’ perceived threat of the virus and their perceived benefit of study participation, but this association was not pronounced. The groups at highest risk of discontinuation mostly comprised pensioners or participants who were single and lived alone. Potential underlying mechanisms could be that pensioners might have had lower digital literacy, and single participants who lived alone may have felt less need to monitor their symptoms due to having less contacts.

This study provides insights into sociodemographic characteristics and vaccination status as potential determinants of decentralized trial continuation, enabled by the large study size and the relative completeness of data. However, while many retention strategies were applied during the COVID-RED trial, we could not robustly assess the effectiveness of these strategies due to the lack of an experimental study design for this purpose. As such, essential information, which could be used to study the effect of the interventions was not collected. Moreover, it would have been worthwhile to investigate more variables as potential determinants, such as participants’ lifestyle and study experiences (eg, the number of alerts received). Finally, as the multivariable logistic regression model was developed ad-hoc during the trial as an exploratory tool, precision and accuracy were not prioritized during model development. Future studies could consider to pre-specify the development of retention models, giving special attention to model performance and potential methodological issues such as limited subgroup sizes.

With this study taking place during an ongoing pandemic when the epidemiological context was constantly changing, results may not translate directly to other settings. On the one hand, participants may have been more engaged in the study because of SARS-CoV-2’s impact on society, and because lockdowns allowed for more free time to comply with study procedures. On the contrary, the virus’ impact on daily life may have made participants more fatigued about the topic and therefore less likely to stay engaged. The study can still serve as a starting point and an inspiration for future decentralized clinical trials conducted in different circumstances. Further research on the topic can help inform effective retention strategies that optimize study resources.

## Conclusion

In this study, we present potential strategies to increase retention and prevent discontinuation in a decentralized clinical trial, and factors potentially associated with a higher risk of dropout. The study was conducted in an unprecedented setting, during the COVID-19 pandemic. Results may help design retention strategies in decentralized clinical trials in the future, although more research is needed to fully understand the drivers of discontinuation in various settings.

## Potential Competing Interests

Drs Zwiers, Veen, Brakenhoff, Goodale, Klaver, Hage, Willigen, and Grobbee are current or previous employees of Julius Clinical. Drs Goodale and Cronin are previous employees of Ava AG. Dr Mitratza is a current employee of P95 CVBA.
