# Supplementary material for: Increasing Retention in a Large-Scale Decentralized Clinical Trial: Learnings From the COVID-RED Trial
Source: Mayo Clin Proc Digit Health. 2025 Sep 9;3(4):100264. doi: 10.1016/j.mcpdig.2025.100264 (PMC12514562; doi:10.1016/j.mcpdig.2025.100264)
Supplement: Supplemental Material [file mmc1.pdf]

Supplementary Materials

Supplement to: *"Increasing retention in a large-scale decentralised clinical trial: learnings from the COVID-RED trial"*.

Supplemental Table 1. Results from multivariable logistic regression model for discontinuation before the start of period 2

	Coefficient	Standard Error	p-value
Intercept	0·139	0·033	<·001*
Age	0·003	0·000	<·001*
Sex (reference group = male)	-0·006	0·006	0·34
Received COVID-19 vaccination	-0·204	0·006	<·001*
**Education**			
Primary education	Reference		
Lower vocational education	0·023	0·033	0·49
Lower general secondary education	0·001	0·031	0·97
Higher general secondary education	0·006	0·032	0·85
Higher vocational education	0·017	0·031	0·57
Higher professional education	0·021	0·031	0·49
University education	0·027	0·031	0·38
No education	-0·005	0·043	0·91
Other education	0·083	0·039	0·034*
**Employment**			
Working at least 80% of full-time	Reference		
Working <80% of full-time	0·012	0·008	0·13
Full-time housekeeper	0·013	0·014	0·35
Full-time student	0·066	0·014	<·001*
Pensioner	0·051	0·012	<·001*
Job-seeker	0·037	0·018	0·040*
(partially) incapacitated	0·037	0·011	0·001*
Rentier	0·020	0·038	0·60
Other	0·031	0·018	0·081
**Living situation**			
Living with partner	Reference		
In a relationship but living alone	-0·026	0·014	0·067
Single and living alone	-0·011	0·008	0·18
Single and living with parents/children	-0·022	0·011	0·055
Living with flatmates	-0·034	0·013	0·010*

*Indicates a p-value <0·05

Supplemental Table 2. Results from multivariable logistic regression model for discontinuation before the start of period 2 with all significant predictors and first-order interactions included

	Coefficient	Standard Error	p-value
Intercept	0·064	0·025	0.010*
Age	0·006	0·000	<·001*
Received COVID-19 vaccination	-0·038	0·026	0.14
**Employment**			
Working at least 80% of full-time	Reference		
Working <80% of full-time	-0·041	0·035	0·24
Full-time housekeeper	-0·083	0·062	0·15
Full-time student	0·046	0·062	0·45
Pensioner	0·032	0·110	0·77
Job-seeker	0·038	0·075	0·62
(partially) incapacitated	-0·052	0·056	0·35
Rentier	-0·619	0·320	0·053
Other	0·065	0·068	0·33
**Living situation**			
Living with partner	Reference		
In a relationship but living alone	-0·015	0·057	0·79
Single and living alone	-0·036	0·034	0·28
Single and living with parents/children	0·075	0·042	0·073
Living with flatmates	0·071	0·046	0·12
**Interaction terms**			
Age × Received COVID-19 vaccination	-0.004	0.001	<·001*
Age × Working <80% of full-time	0·001	0·001	0·16
Age × Full-time housekeeper	0·003	0·001	0·046*
Age × Full-time student	-0·000	0·002	0·99
Age × Pensioner	-0·000	0·002	0·77
Age × Job-seeker	-0·001	0·002	0·65
Age × (partially) incapacitated	0·002	0·001	0·12
Age × Rentier	0·009	0·005	0·060
Age × Other employment	-0·001	0·001	0·49
Age × In a relationship but living alone	-0.000	0.001	0.88

Age × Single and living alone	-0.000	0.001	0.83
Age × Single and living with parents/children	-0.003	0.001	<·001*
Age × Living with flatmates	-0.004	0.001	0.001*
Working <80% of full-time × Received COVID-19 vaccination	0.009	0.016	0.59
Working <80% of full-time × In a relationship but living alone	-0.004	0.045	0.94
Working <80% of full-time × Single and living alone	-0.007	0.026	0.80
Working <80% of full-time × Single and living with parents/children	0.039	0.032	0.22
Working <80% of full-time × Living with flatmates	-0.018	0.043	0.68
Full-time housekeeper × Received COVID-19 vaccination	-0.022	0.029	0.46
Full-time housekeeper × In a relationship but living alone	-0.086	0.092	0.35
Full-time housekeeper × Single and living alone	-0.051	0.048	0.29
Full-time housekeeper × Single and living with parents/children	-0.005	0.060	0.93
Full-time housekeeper × Living with flatmates	0.015	0.092	0.87
Full-time student × Received COVID-19 vaccination	-0.057	0.027	0.035*
Full-time student × In a relationship but living alone	-0.064	0.064	0.32
Full-time student × Single and living alone	0.099	0.047	0.034*
Full-time student × Single and living with parents/children	0.010	0.043	0.81
Full-time student × Living with flatmates	0.012	0.043	0.78
Pensioner × Received COVID-19 vaccination	0.083	0.027	0.002*
Pensioner × In a relationship but living alone	-0.046	0.061	0.45
Pensioner × Single and living alone	-0.005	0.030	0.87
Pensioner × Single and living with parents/children	0.026	0.096	0.78
Pensioner × Living with flatmates	0.207	0.086	0.016*
Job-seeker × Received COVID-19 vaccination	0.031	0.037	0.40
Job-seeker × In a relationship but living alone	0.062	0.079	0.43
Job-seeker × Single and living alone	0.021	0.045	0.65
Job-seeker × Single and living with parents/children	-0.002	0.061	0.97
Job-seeker × Living with flatmates	0.060	0.070	0.39
(partially) incapacitated × Received COVID-19 vaccination	0.007	0.023	0.75
(partially) incapacitated × In a relationship but living alone	-0.031	0.051	0.54
(partially) incapacitated × Single and living alone	0.009	0.027	0.74
(partially) incapacitated × Single and living with parents/children	0.019	0.047	0.68
(partially) incapacitated × Living with flatmates	0.014	0.056	0.80
Rentier × Received COVID-19 vaccination	0.001	0.088	0.99
Rentier × In a relationship but living alone	-0.151	0.163	0.36
Rentier × Single and living alone	0.186	0.092	0.043*
Rentier × Single and living with parents/children	0.963	0.380	0.011*
Rentier × Living with flatmates	0.803	0.277	0.004*
Other employment × Received COVID-19 vaccination	0.037	0.037	0.31
Other employment × In a relationship but living alone	-0.064	0.075	0.39
Other employment × Single and living alone	-0.014	0.047	0.81
Other employment × Single and living with parents/children	0.002	0.061	0.97
Other employment × Living with flatmates	-0.073	0.068	0.28
In a relationship but living alone × Received COVID-19 vaccination	0.033	0.029	0.26
Single and living alone × Received COVID-19 vaccination	0.048	0.017	0.004*
Single and living with parents/children × Received COVID-19 vaccination	0.022	0.023	0.33
Living with flatmates × Received COVID-19 vaccination	0.043	0.027	0.11

*Indicates a p-value <0·05
